# Positional effects on the distributions of ventilation and end-expiratory gas volume in the asymmetric chest—a quantitative lung computed tomographic analysis

**DOI:** 10.1186/s40635-018-0175-4

**Published:** 2018-04-10

**Authors:** Gustavo A. Cortes-Puentes, Kenneth E. Gard, Alexander B. Adams, David J. Dries, Michael Quintel, Richard A. Oeckler, Luciano Gattinoni, John J. Marini

**Affiliations:** 1 Department of Pulmonary and Critical Care Medicine, Mayo Clinic, 200 First Street SW, Rochester, MN 55905 USA; 2 Department of Pulmonary and Critical Care Research, Regions Hospital, Office E3844, 640 Jackson Street, St. Paul, MN 55101 USA; 3 Department of Surgery, University of Minnesota, Regions Hospital, Office E3844, 640 Jackson Street, St. Paul, MN 55101 USA; 4 Department of Anesthesiology, Emergency and Intensive Care Medicine, University of Göttingen, Robert-Koch-Str. 40, 37075 Göttingen, Germany; 5 Department of Pulmonary and Critical Care Medicine, University of Minnesota, Regions Hospital, MS11203B, 640 Jackson Street, St. Paul, MN 55101 USA

**Keywords:** Pleural effusion, Tidal recruitment, Body position, Prone, Quantitative computed tomography, Analysis of aeration, Chest wall asymmetry, Functional residual capacity

## Abstract

**Background:**

Body positioning affects the configuration and dynamic properties of the chest wall and therefore may influence decisions made to increase or decrease ventilating pressures and tidal volume. We hypothesized that unlike global functional residual capacity (FRC), component sector gas volumes and their corresponding regional tidal expansions would vary markedly in the setting of unilateral pleural effusion (PLEF), owing to shifting distributions of aeration and collapse as posture changed.

**Methods:**

Six deeply anesthetized swine underwent tracheostomy, thoracostomy, and experimental PLEF with 10 mL/kg of radiopaque isotonic fluid randomly instilled into either pleural space. Animals were ventilated at V_T_ = 10 mL/kg, frequency = 15 bpm, I/E = 1:2, PEEP = 1 cmH_2_O, and FiO_2_ = 0.5. Quantitative lung computed tomographic (CT) analysis of regional aeration and global FRC measurements by nitrogen wash-in/wash-out technique was performed in each of these randomly applied positions: semi-Fowler’s (inclined 30° from horizontal in the sagittal plane); prone, supine, and lateral positions with dependent PLEF and non-dependent PLEF.

**Results:**

No significant differences in total FRC were observed among the horizontal positions, either at baseline (*p* = 0.9037) or with PLEF (*p* = 0.58). However, component sector total gas volumes in each phase of the tidal cycle were different within all studied positions with and without PLEF (*p* = < .01). Compared to other positions, prone and lateral positions with non-dependent PLEF had more homogenous V_T_ distributions among quadrants (*p* = .051). Supine position was associated with most dependent collapse and greatest tendency for tidal recruitment (48 vs ~ 22%, *p* = 0.0073).

**Conclusions:**

Changes in body position in the setting of effusion-caused chest asymmetry markedly affected the internal distributions of gas volume, collapse, ventilation, and tidal recruitment, even though global FRC measurements provided little indication of these potentially important positional changes.

**Electronic supplementary material:**

The online version of this article (10.1186/s40635-018-0175-4) contains supplementary material, which is available to authorized users.

## Background

Monitoring the respiratory system mechanics of the intubated patient is mandatory in order to assure safety and effectiveness of mechanical ventilation. Although the clinical target of primary interest is the lung, which typically is influenced by gravity and mechanically heterogeneous [1], clinicians have traditionally relied upon the pooled data derived from delivered and expired gas flows as well as the circuit (airway) pressure applied across the series-coupled lung and chest wall. The chest wall can be considered as comprised of three distinct but interactive sectors: the left hemi-thoracic, right hemi-thoracic, and abdominal compartments.

Chest wall characteristics often influence decisions made to increase or decrease positive end-expiratory pressure (PEEP) and tidal volume [2], and these are altered by body position [3]. A decline in functional residual capacity (FRC) and a global increase in chest wall stiffness are well documented to result in the transition from upright to horizontal supine posture within the vertical (sagittal) plane [3, 4]. Much less is known about *regional* volume changes that occur within the lung during recumbent rotations along the unchanging horizontal axis, especially in the setting of non-symmetrical chest disease.

Recently, we showed by modified gas dilution methodology that FRC and transpulmonary pressure actually change little as position is altered within the horizontal 0° axis among supine, prone, and lateral decubitus orientations [3]. This indifference of total gas volume to posture was observed despite highly non-symmetric pleural topography caused by a large unilateral effusion. In the present study, we used quantitative computed tomography (CT) to explore the influence of these same positions and non-symmetries on *regional* ventilations and resting lung volumes within ventral and dorsal anatomic sectors of the ipsilateral and contralateral lungs. We hypothesized that unlike global FRC, component sector gas volumes and their corresponding regional tidal expansions would vary markedly, owing to shifting *distributions* of aeration and collapse as posture varied.

## Methods

### Animal preparation

The protocol was approved by the Animal Care and Use Committee of Regions Hospital (St. Paul, MN, USA). Six young healthy male and female Yorkshire pigs, mean weight 30.3 ± 3.8 kg, were premedicated with intramuscular Telazol/xylazine (2.2 and 6.6 mg/kg, respectively). After tracheostomy and intubation with a cuffed endotracheal tube, pigs received a continuous flow (0.08–2 L/min) of isoflurane—50% + nitrous oxide inhalational mixture for the remainder of the instrumentation period. We performed femoral venous/arterial catheter placements, suprapubic cystostomy in all animals. Inhalational anesthesia was slowly discontinued over ~ 30 min and replaced by a titrated intravenous (IV) drip infusion of Telazol, ketamine, and xylazine to maintain deep anesthesia, adjusted as indicated by intermittent reflex testing and continuous bispectral analysis (BIS; Covidien, Carlsbad, CA, USA) to assure that no breathing efforts could be observed in the airway pressure tracing at any time [5]. During preparation and throughout the study, all animals were paralyzed with boluses of pancuronium 0.1 mg/kg and mechanically ventilated with an Engström Carestation™ ventilator (GE Healthcare, Madison, WI): constant flow volume control ventilation, tidal volume (V_T_) 10 mL/kg, respiratory rate titrated to an end-tidal carbon dioxide (CO_2_) of 30–40 mmHg at baseline, inspiratory to expiratory ratio (I:E) of 1:2, PEEP of 1 cmH_2_O (PEEP1), and fraction of inspired oxygen (FiO2) of 0.5. A chest tube (Cook Medical, Bloomington, IN, USA) was inserted with a cephalad orientation at the level of the seventh or eighth intercostal space; air entering the pleural space during the procedure was subsequently aspirated. In three randomly selected animals, thoracostomies were performed on the right side, and in the remainder, thoracostomy was performed on the left side. When required by the protocol, 10 mL/kg of saline at body temperature was instilled to simulate pleural effusion (PLEF).

Oxygen saturation, end-tidal CO_2_, airway pressure, arterial pressure, and heart rate were continuously monitored. Intravenous fluid administration consisted of an initial bolus of 12 mL/kg normal saline, followed by a 3 mL/kg/h replacement and additional 4 mL/kg boluses if mean arterial pressure (MAP) fell to < 60 mmHg. Body temperature was maintained between 36 and 37 °C with heating pads and a Gaymar™ Heat Pump. Respiratory system mechanics and end-expiratory gas lung volume (FRC) responses to PLEF and position changes were recorded. Each animal also underwent sequential thoracic CT aeration analysis under all experimental conditions. Using cinematic scanning capabilities of the CT equipment, images of the first eight cephalad supra-diaphragmatic slices were obtained at 0.4 s intervals during both end-inspiration and end-expiration [6]. At the end of each experiment, animals were euthanized by rapid injection of Euthasol®.

### Experimental protocol

Respiratory system mechanics and FRC were evaluated in response to unilateral PLEF and positioning. Volume-controlled ventilation (VCV) settings remained unmodified during the experimental protocol except during recruitment maneuvers after position changes, which were performed using ten breaths of pressure-controlled ventilation (PCV) with an inspiratory pressure of 40 cmH_2_O and PEEP = 20 cmH_2_O [7]. FRC was measured using a ventilator-integrated modified nitrogen wash-in/wash-out method (Engström Carestation™, GE Healthcare, Madison, WI, USA) [8]. A PEEP of 1 cmH_2_O (PEEP1) is a technical requirement when FRC is measured using the proprietary modified wash-in/wash-out technique (GE Healthcare, Madison, WI, USA) and served as the least value for end-expiratory airway pressure [8]. FRC was measured in five positions, applied in random order, before (baseline) and after PLEF instillation: semi-Fowler’s (inclined 30° from horizontal in the sagittal plane), prone, supine, right lateral, and left lateral. FRC measurements during semi-Fowler’s position were aimed at confirming that the gas dilution methodology we utilized was able to detect the expected differences in lung volumes between horizontal and more upright conditions.

An isotonic radio-opaque solution was instilled into the pleural cavity via chest tube to generate a PLEF (10 mL/kg); this solution was prepared as follows: 100 mL of iopromide at a concentration of 300 mg iodine per milliliter (mgI/mL) (Ultravist™ Injection 300 mgI/mL, Bayer HealthCare, Germany) + 800 mL of 0.9% saline + 100 mL of deionized water. This contrasted fluid displays an attenuation coefficient CT number range from 300 to 700 Hounsfield units (HU) with a peak at 560 HU. Positional effects on end-expiratory gas and tissue volumes as measured by sequential thoracic computed tomography (CT) aeration analysis were evaluated in all horizontal positions—prone, supine, right lateral, and left lateral after PLEF. All measurements were obtained after a 10-min stabilization period in a given tested position. Vital signs were monitored during the entire experiment.

### Computed tomography measurements and lung volume analysis

Helical images of the chest were obtained with a 64-slice CT scanner (LightSpeed™ VCT, GE Healthcare, Milwaukee, WI) using the following parameters: 120 peak kilovoltage (kVp), 575 mA, collimation of 64 × 0.625 mm, pitch of 1, and gantry rotation time of 0.5 s. Images were reconstructed using the “Standard” kernel with a slice thickness of 2.5 mm and reconstruction field of view from 220 to 280 mm (pixel size from 0.43 to 0.55 mm). All animals underwent CT at PEEP = 1 cmH_2_O. In all animals, lung CT scans were acquired at each step at end-expiration (EE) and end-inspiration (EI), by taking CT images every 0.4 s with a 0.5-s cycle pause, ensuring measurement at EI and EE were captured during their cycle peaks. After an initial whole lung scan to determine the position of the diaphragm at end-inspiration and end-expiration, the first eight supradiaphragmatic slices were selected for cinematic CT (CINE) [8].

#### Image analysis

**Fig. 1:**
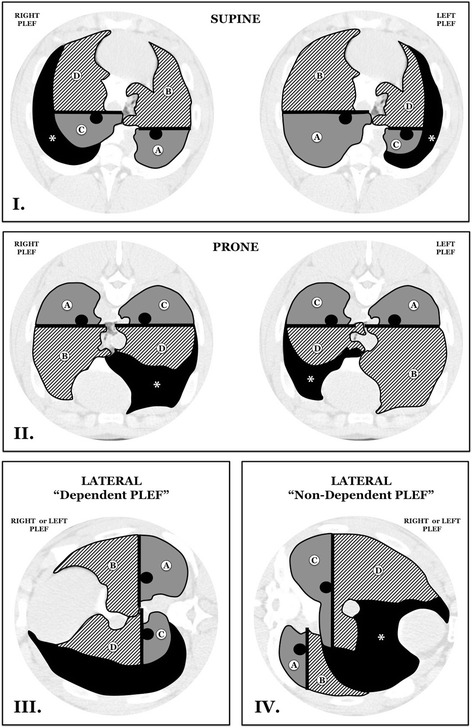
Schematic images in all horizontal positions and their respective anatomical and regional demarcations. **I** *Supine* and **II** *prone*, where quadrants were defined as non-PLEF dorsal (A), non-PLEF ventral (B), PLEF dorsal (C), and PLEF ventral (D). **III** *Lateral position with* “*dependent pleural effusion*,” where quadrants were defined as non-PLEF, non-dependent dorsal (A); non-PLEF, non-dependent ventral (B), PLEF-dependent dorsal (C), and PLEF-dependent ventral (D). **IV** *Lateral position with* “*non-dependent pleural effusion*,” where quadrants were defined as non-PLEF, dependent dorsal (A); non-PLEF, dependent ventral (B); PLEF, non-dependent dorsal (C); and PLEF non-dependent ventral (D). *PLEF* pleural effusion. Asterisk denotes anatomical distribution of PLEF. Note that the quadrant-demarcating (anatomic) ventral surfaces of the left and right main bronchi shifted in the two lateral positions. Also refer to Additional file 1 for representative chest CT images of each studied condition

End-expiratory and end-inspiratory gas and tissue volumes of the lung were computed using a well-established approach based on the tight correlation between CT attenuation and physical density of the tissue, as previously described by Gattinoni and colleagues [6]. In both CT acquisition phases (EE, EI), lung parenchyma was manually delineated from the chest wall, PLEF, and mediastinal structures. For the purpose of our analysis, “ventral” and “dorsal” regions were separated anatomically by a horizontal line across the ventral border of each main stem bronchus (refer to Fig. 1 for the schematic representation of lung boundaries and segmental nomenclature used during the CT analysis). By using “Osiris” software (Digital Imaging Unit, University Hospital of Geneva, Switzerland), regions of interest were selected on both right and left sides, and non-parenchymal pulmonary structures were eliminated from the regional analysis [6]. Lung tissue was sub-classified based on CT attenuation changes as follows: normally aerated (CT values between − 500 HU and − 900 HU); poorly aerated (between − 100 HU and − 500 HU); non-aerated or collapsed (between − 100 HU and + 100 HU); and hyper-inflated (lower than − 900 HU) [6, 9, 10]. To compute tidal lung recruitability, the difference in weight of not inflated lung tissue at each tidal cycle (EI, EE) was normalized by the total lung weight measured at end-expiration (EE) [6].

### Statistics

Within condition effects were estimated using linear regression. Between condition (with and without PLEF for FRC measurements) and between position effects were estimated by including the appropriate interaction term in a linear regression model adjusted for correlation of measurements within pig. In addition, we used Tukey correction for post hoc multiple comparisons. A *p* value < 0.05 was considered significant.

## Results

A set of schematic CT images in all horizontal positions and their respective anatomical and regional demarcations (“quadrants”) are illustrated in Fig. 1. Nomenclature and labeling were maintained during the regional analyses of aeration. Representative chest CT images in all studied conditions are included in Additional file 1.

### FRC response to changes in body position

**Fig. 2:**
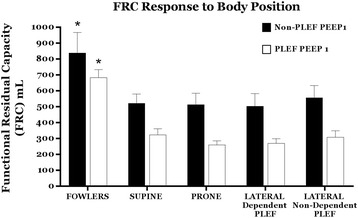
FRC response to body position. No differences in FRC were observed among the four horizontal positions (left, prone, right, and supine) with or without PLEF at PEEP1. Semi-Fowler’s position exhibited significantly higher FRC compared to all horizontal positions with and without PLEF (asterisk denotes statistical significance). Means and standard deviations are shown. *BSL* baseline, *PEEP* positive end-expiratory pressure, *PLEF* pleural effusion, *FRC* functional residual capacity

FRC values are displayed in Fig. 2. Although PLEF significantly reduced FRC across all conditions when compared to baseline (*p* < 0.05), no significant differences in FRC were observed among the horizontal positions (supine, prone, right lateral, and left lateral) at baseline (*p* = 0.9) or with PLEF (*p* = 0.06). FRC significantly increased with semi-Fowler positioning compared to each of the horizontal positions (*p* = < 0.01).

### Effect of body positioning on total gas volume and ventilation distribution

**Fig. 3:**
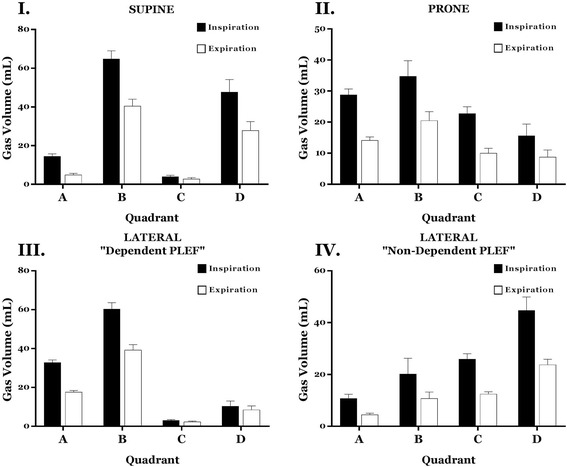
**I**–**IV** Total gas volumes at end-inspiration and end-expiration for each position. Please refer to Fig. 1 for nomenclature. Total gas volumes at end-inspiration and end-expiration for each position are displayed. Total gas volume in each phase of the tidal cycle was significantly different *within* all positions with and without PLEF. Means and standard deviations are shown. *PLEF* pleural effusion

The tidal differences in gas volume (mL) contained in normally aerated, poorly aerated, and hyper-inflated lung tissue were analyzed for each quadrant in four body positions in relationship to the presence of unilateral (*right or left*) PLEF (see Fig. 1 for nomenclature of analysis). Total gas volumes at end-inspiration and end-expiration for each position are displayed in Fig. 3. Total gas volume in each phase of the tidal cycle (EI, EE) was significantly different *within* all positions with and without PLEF (*p* = < .01).

**Fig. 4:**
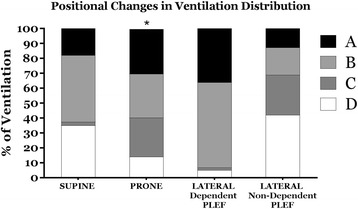
Distribution of tidal ventilation. Please refer to Fig. 1 for nomenclature. Tidal changes in regional gas volume were significantly different in the supine and lateral positions with dependent and non-dependent PLEF. Prone position (asterisk), however, showed a more homogeneous gas volume distribution among quadrants compared to other horizontal positions

The percentage of distribution of tidal differences in gas volume (tidal ventilation) is displayed in Fig. 4. Significant differences in tidal changes in regional gas volumes were observed between the quadrants in the supine position (*within* position difference, *p* = < .001, Fig. 4). In the prone position, gas volume distribution was more homogeneous among the quadrants (*between* position difference, *p* = .051, Fig. 4). Overall, tidal changes in regional gas volumes were distributed differently *between* supine and prone positions (*p* = < .0001).

Tidal changes in gas volume were significantly different among the quadrants within both the “lateral position with dependent PLEF” (*within* position difference, *p* = < .0001, Fig. 4) and “lateral position with non-dependent PLEF” (*within* position difference, *p* = .004, Fig. 4). Additionally, tidal changes in regional gas volumes were different *between* lateral positions with non-dependent and dependent PLEF (*p* = < .0001); however, the pattern of distribution was more homogeneous among the quadrants when the PLEF was in the non-dependent position.

### Effect of body positioning on tidal opening and closure

**Fig. 5:**
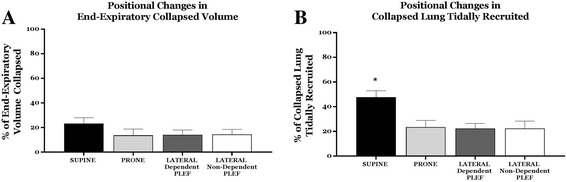
Positional changes in end-expiratory volume collapsed and tidal recruitment of the collapsed lung. The average percentage of end-expiratory volume collapsed did not change as horizontal positions varied (**a**). Compared to other horizontal positions, supine showed a significantly higher percentage of tidally recruited collapsed lung volume (**b**). Means’ percentage and standard deviations are shown

There was no significant difference in the average percentage of end-expiratory volume collapsed among the horizontal positions studied (*p* = 0.4, Fig. 5a). Tidal *changes* in collapsed lung were also analyzed (Fig. 5b). Interestingly, approximately 48% of the collapsed lung volume at the end-expiration in the supine position opened at the end of inspiration (*between* position difference, supine vs. other horizontal positions *p* = 0.007) versus ~ 22% in each of the other positions (prone, lateral position with dependent pleural effusion and lateral position with non-dependent pleural effusion, *p* = > .05).

Position changes did not influence measured variables of cardiovascular and respiratory status (heart rate, MAP, and oxygen saturation) during any of the tested conditions. As described in our previous work during the same conditions at PEEP1 [3], transpulmonary pressure (like global FRC) was insensitive to mechanical asymmetry and to changes in regional gas volume caused by varying position [3]. Representative CT images of each of the studied positions are available in Additional file 1.

## Discussion

The key observations resulting from this study can be summarized as follows.

In the setting of large unilateral pleural effusion and pigs passively ventilated and rotated randomly (four 90° shifts) along the horizontal (recumbent) 0° axis:

1. Prone and lateral positioning radically alters the internal distributions of end-expiratory gas volume and ventilation observed supine but leaves relatively unaffected the global measures of FRC.
2. Re-positioning from supine into the prone, and effusion-dependent and effusion-non-dependent lateral postures did not markedly influence the total amount of end-expiratory collapse.
3. The position-associated changes of ventilation distribution paralleled those of end-expiratory lung volume in all orientations.
4. Prone positioning helped even the regional distributions of gas volume and ventilation.
5. Superior positioning of the effusion dramatically improved the relative proportions of both FRC and ventilation delivered to the ipsilateral lung.
6. Among the four horizontal positions, the supine orientation is associated with most dependent collapse and greatest tendency for tidal recruitment.

Changing body position can alter the distributions of gas volume, perfusion, and collapse, as demonstrated in published studies of prone versus supine positioning [6, 11–14]. At the bedside, these alterations are manifest in altered gas exchange and symptoms that accompany repositioning [12]. Without exception, positioning studies have addressed healthy lungs or those with relatively symmetrical abnormality, such as experimental acute lung injury. Less documentation of position-related changes of mechanics is available for the setting of non-symmetrically distributed disease. In previous work that addressed a model of unilateral effusion [3], we demonstrated that neither the transpulmonary pressure nor the functional residual capacity (FRC) changes markedly with rotational shifts in position that occur around the 0° horizontal axis [3]. Such global measurements blend information that originates within a lung that may be asymmetrically affected. Simple visual inspection of CT images (Additional file 1), however, makes it clear that regional aeration may be remarkably different in varied positions, demonstrating the capacity of interdependence of the integrated respiratory system to maintain aerated lung volume. The work presented here confirms and quantifies that impression in a model of unilateral pleural effusion by demonstrating the significant distributional changes of regional gas volume (Fig. 3), ventilation (Fig. 4), and both static and dynamic tidal collapse that occur during positional rotations around the 0° (horizontal) axis.

Although ours is not a lung injury model, we demonstrate that prone repositioning helps even the regional distributions of aeration and ventilation when compared to supine or lateral decubitus positions, even in this setting of highly unilateral mechanical abnormality. Moreover, prone positioning appears to reduce the fraction of collapsed tissue that opens and closes within each tidal breath when compared to supine (Fig. 5b). These alterations would appear to reinforce the generally agreed-upon elements of a lung protective approach to ventilation that includes prone positioning [15–17].

As expected, turning the subject with unilateral effusion into the ipsilateral dependent and ipsilateral nondependent orientations radically modifies the distributions of aeration and ventilation. One relatively surprising aspect of our data is that the regional distribution of ventilation paralleled the regional distribution of aeration at FRC (Figs. 3 and 4). Superior positioning of the ipsilateral effusion improved the relative ventilation of those quadrants (Fig. 4, *lateral position with non-dependent PLEF*).

The near equivalence of FRC among the 0° positions suggests that the total volume of collapsed tissue would be similar as well, i.e., independent of body orientation. Yet, our data indicate that at least for this model, the supine position predisposes to greater tidal recruitment and collapse then do the other horizontal conditions. The reason for this would appear to be that in the lateral positions, the relatively open superior lung receives disproportionate ventilation, whereas the dependent lung (where compressive forces are greater and collapse predominates) is relatively deprived of ventilation and therefore of the peak transpulmonary pressures needed to reopen collapsed tissue [3, 18–21].

### Limitations

We acknowledge that both our model and analysis are open to question in several areas. We made two methodological assumptions that we believe to be reasonable. First, because of the highly labor intensive nature of manually selecting regions of interest for quantitative analysis, we restricted attention to a representative tranche of tissue in the transverse plane, rather than the entire lung. Because we and others have shown this approximation to be representative [8, 9], this compromise is now standard analytical practice [9]. Second, rather than using anatomical boundaries, we sectioned the acquired CT images into quadrants defined as combinations of dependent and non-dependent, ventral and dorsal portions. This choice was made because fluid was instilled into the left pleural compartment in three animals and into the right pleural compartment in the remaining three. The aeration and ventilation patterns followed exactly similar trends in both groups, prompting the current analysis. Not to have done so would have required an unnecessary doubling of our sample size.

Although this large animal representation of nonsymmetrical chest disease is appealing as one in which the compressive forces created by the large effusion cause disproportionately ipsilateral lung collapse and rib cage expansion, it must be emphasized that the thoracic anatomies of the pig and human are not equivalent [22]. These anatomical differences extend to the flexibility of the diaphragm and abdomen. Conceivably, interdependence among the thoracic and abdominal compartments that comprise the chest wall may be better developed in swine [22]. Another potential concern is that the normal orientations of pig and human are prone and upright, respectively. The amplitude of the data signals are certain to be subject to the volume of instilled pleural fluid and the application of PEEP, as well as by the nature of the model itself. Although this model produced regions of tissue collapse and recruitability, we did not study a preparation in which the chest wall itself was deformed (other than by variations of position) or the lung directly injured. Because the chest wall of the pig is relatively flexible and the liquid freely mobile, relatively large positional changes of regional ventilation and aeration are to be expected. What was unexpected, however, was that such major distributional changes were not accompanied by alterations of FRC determined by gas dilution (Fig. 2).

Finally, CT analysis of aeration was only performed at PEEP1, and higher levels of PEEP could certainly influence our results. However, in our previous experience with a similar experimental model [3], no significant differences in FRC were seen among the supine, prone, right lateral, and left lateral variations of horizontal position, with or without pleural effusion at either PEEP1 or PEEP of 10 cmH2O (PEEP10). Additionally, in transitioning from PEEP1 to PEEP10 (with or without PLEF), no changes in P_TP_ driving pressure (DP_TP_, the difference between end-inspiratory and end-expiratory P_TP_) were observed among all horizontal positions, although end-expiratory P_TP_ increased from a negative pressure to a positive or near-zero pressure as position changed in the horizontal axis [3]. Therefore, since DP_TP_ does not seem to change as body position is adjusted in the horizontal plain, we hypothesize that even though positional changes in both end-expiratory volume collapsed and tidal recruitment of collapsed lung may decrease as higher PEEP values are applied, the segmental *distribution* of tidal ventilation observed at PEEP1 could potentially be maintained at higher levels of PEEP. In such a mechanically asymmetrical setting, however, it is possible that higher PEEP might accentuate or attenuate distributional heterogeneity even as it lessens the overall amplitude of tidal recruitment.

Although we believe that the physiologic principles elucidated here are qualitatively valid, observations made in this animal model clearly would need to be extended into studies of the relevant clinical state before the implications are accepted for medical use. Assuming that more homogeneous regional distribution of tidal ventilation parallels betters gas exchange and regional pulmonary mechanics (regional DP_TP_), our results suggest that mechanically ventilated patients with a large unilateral PLEF would experience lower end-expiratory volume collapsed and more homogeneous ventilation while in *prone position* and *lateral position with non-dependent PLEF.*

## Conclusions

The data provided in this experimental study of nonsymmetrical chest mechanics demonstrate the potential for alterations of body position to markedly affect the internal distributions of gas volume, collapse, ventilation, and tidal recruitment, even when commonly measured global indicators of transpulmonary pressure and functional residual capacity provide little indication of these important positional changes. Our results underscore the adaptability of interdependence forces as well as the potential value of extending bedside measures of mechanics beyond those based on airway pressure, esophageal pressure, and flow by interrogating internal anatomy and dynamics as well as respiratory gas exchange with techniques such as electrical impedance tomography (EIT) and thoracic ultrasound.

## Additional file

Additional file 1: **Figure S1.** Chest CT images at end-inspiration during supine and prone positions. Radiopaque material indicates the location of the pleural effusion randomly instilled in the right (*n* = 3) or left pleural (*n* = 3) space. **Figure S2.** Chest CT images at end-inspiration during lateral positions with dependent and non-dependent PLEF. Radiopaque material indicates the location of the pleural effusion randomly instilled in the right (*n* = 3) or left pleural (*n* = 3) space. PLEF = pleural effusion. (DOCX 753 kb)

## Abbreviations

BIS: Continuous bispectral analysis
CINE: Cinematic computed tomography
CO_2_: Carbon dioxide
CT: Computed tomography
EE: End-expiration
EI: End-inspiration
EIT: Electrical impedance tomography
FiO2: Fraction of inspired oxygen
FRC: Functional residual capacity
HU: Hounsfield units
I:E: Inspiratory to expiratory ratio
IV: Intravenous
MAP: Mean arterial pressure
PCV: Pressure-controlled ventilation
PEEP: Positive end-expiratory pressure
PEEP1: PEEP of 1 cmH_2_O
PLEF: Pleural effusion
VCV: Volume-controlled ventilation
V_T_: Tidal volume
