# Supplementary material for: Positional effects on the distributions of ventilation and end-expiratory gas volume in the asymmetric chest—a quantitative lung computed tomographic analysis
Source: Intensive Care Med Exp. 2018 Apr 10;6:9. doi: 10.1186/s40635-018-0175-4 (PMC5891440; doi:10.1186/s40635-018-0175-4)

**Additional file 1**

Positional Effects on the Distributions of Ventilation and End-expiratory Gas Volume in the Asymmetric Chest –A Quantitative Lung Computed Tomographic Analysis

Gustavo A. Cortes-Puentes, MD

Kenneth E . Gard, MD

Alexander B. Adams, MPH, RRT

David J. Dries, MD

Michael Quintel, MD

Richard A. Oeckler, MD, PhD

Luciano Gattinoni, MD

John J Marini, MD

*Corresponding Author:*

Gustavo A. Cortes-Puentes, MD

Mayo Clinic, Rochester MN, United States.

Department of Pulmonary and Critical Care Medicine

Mailing address: Mayo Clinic, 200 First Street SW, Rochester, MN 55905 USA

E-mail: [CortesPuentes.Gustavo@mayo.edu](mailto:CortesPuentes.Gustavo@mayo.edu)

**Figure S1: Chest CT Images at End-Inspiration During Supine and Prone positions.** Radiopaque material indicates the location of the pleural effusion randomly instilled in the right (n=3) or left pleural (n=3) space.


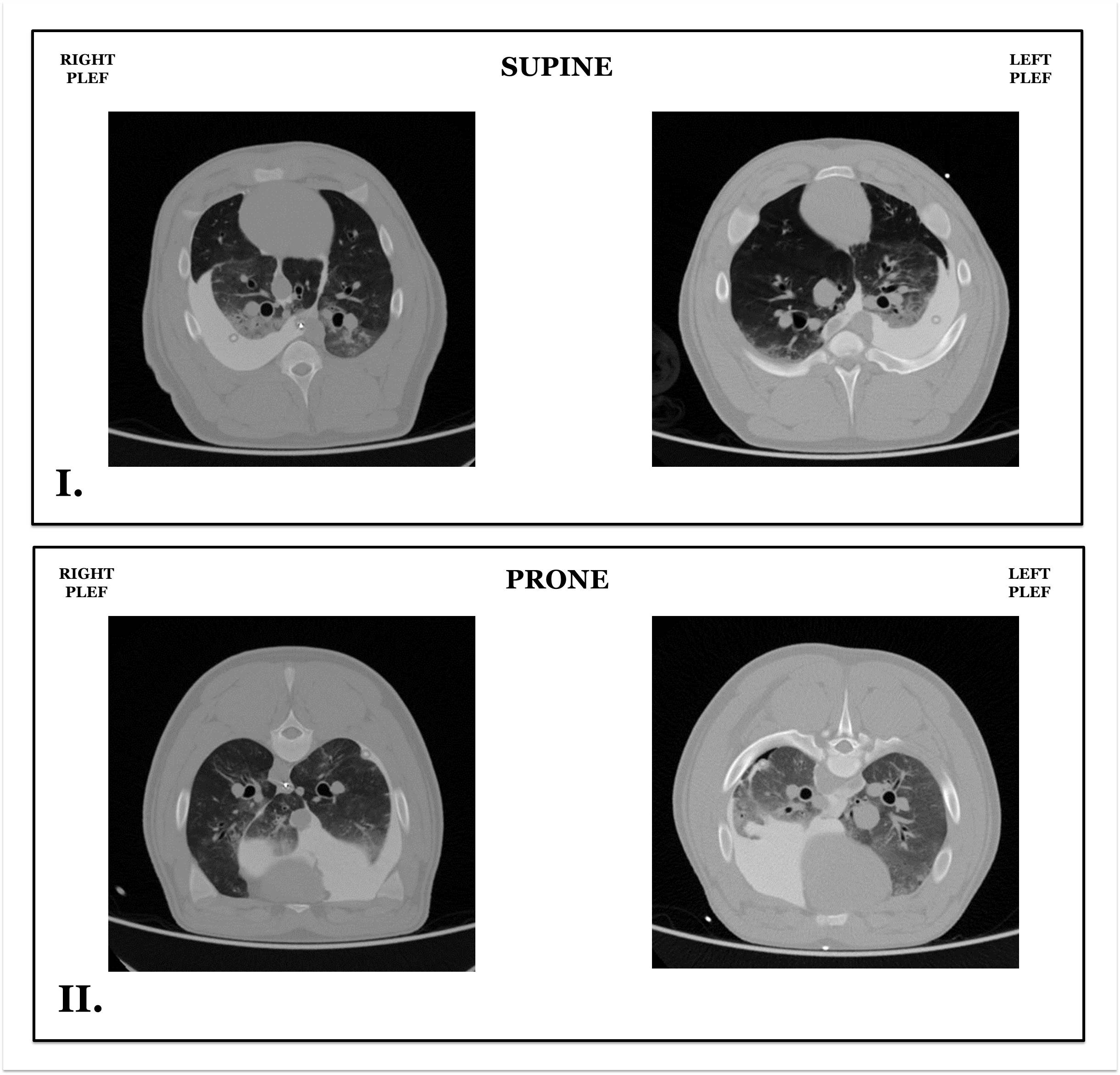


**Figure S2: Chest CT Images at End-Inspiration During Lateral Positions with Dependent and Non-Dependent PLEF.** Radiopaque material indicates the location of the pleural effusion randomly instilled in the right (n=3) or left pleural (n=3) space. PLEF= pleural effusion.


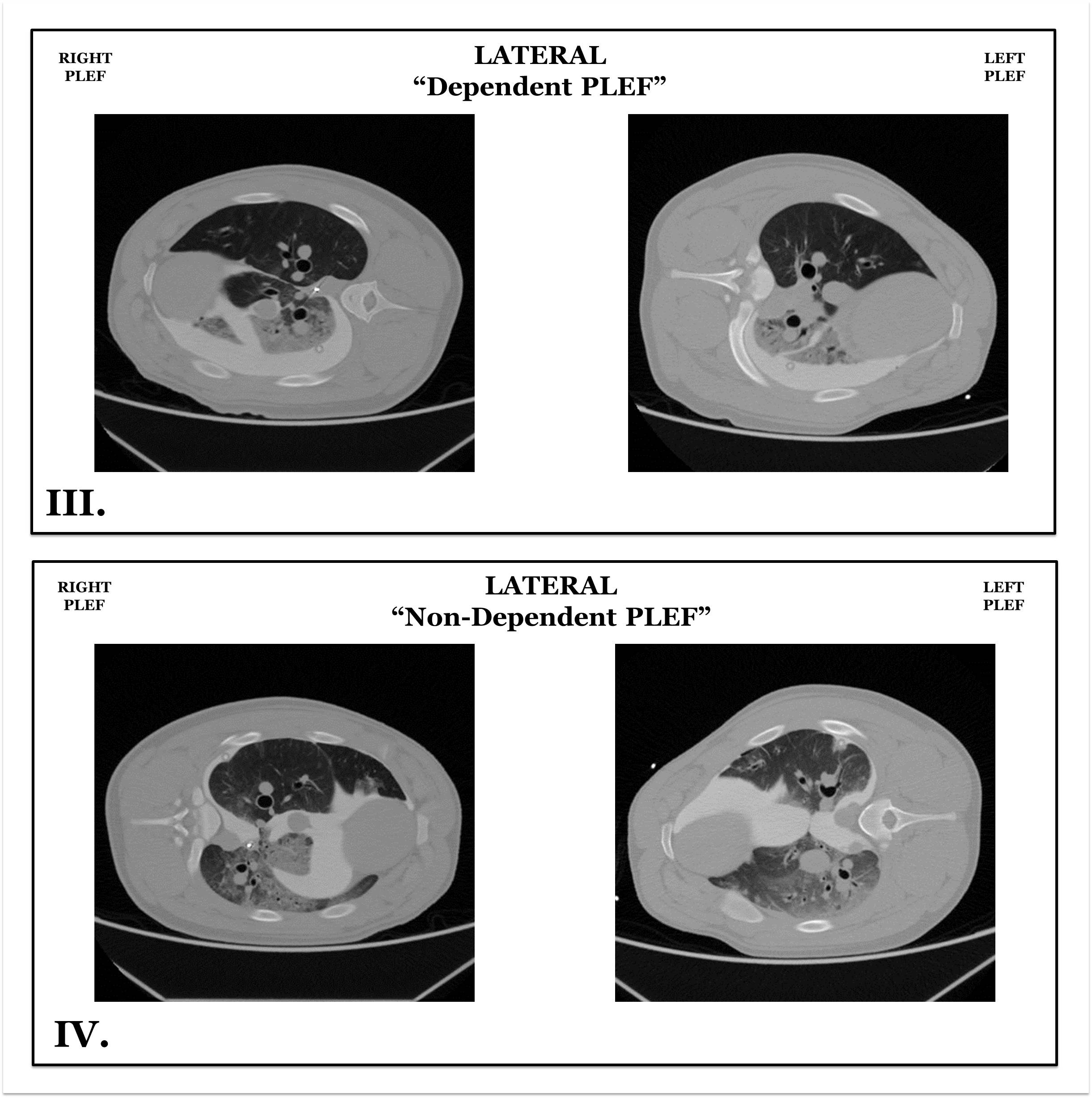

Supplement: Supplementary file 1 — Figure S1. Chest CT images at end-inspiration during supine and prone positions. Radiopaque material indicates the location of the pleural effusion randomly instilled in the right (n = 3) or left pleural (n = 3) space. Figure S2. Chest CT images at end-inspiration during lateral positions with dependent and non-dependent PLEF. Radiopaque material indicates the location of the pleural effusion randomly instilled in the right (n = 3) or left pleural (n = 3) space. PLEF = pleural effusion. (DOCX 753 kb) [file 40635_2018_175_MOESM1_ESM.docx]
